# 4-[(2-Hydr­oxy-1-naphth­yl)methyl­idene­amino]benzoic acid

**DOI:** 10.1107/S1600536808006107

**Published:** 2008-03-07

**Authors:** Mehmet Akkurt, Sema Öztürk Yıldırım, Abdullah Mohamed Asiri, Vickie McKee

**Affiliations:** a Department of Physics, Faculty of Arts and Sciences, Erciyes University, 38039 Kayseri, Turkey; b Chemistry Department, Faculty of Science, King Abdul-Aziz University, P. O. Box 80203, Jeddah 21589, Saudi Arabia; c Department of Chemistry, Loughborough University, Leicestershire LE11 3TU, England

## Abstract

The mol­ecule of the title compound, C_18_H_13_NO_3_, is almost planar, the dihedral angle between the naphthalene and benzene ring systems being 4.04 (6)°. The mol­ecular conformation and packing are stabilized by intra­molecular O—H⋯N and inter­molecular O—H⋯O and C—H⋯O inter­actions.

## Related literature

For background, see: Asiri & Badahdah (2007 ▶).
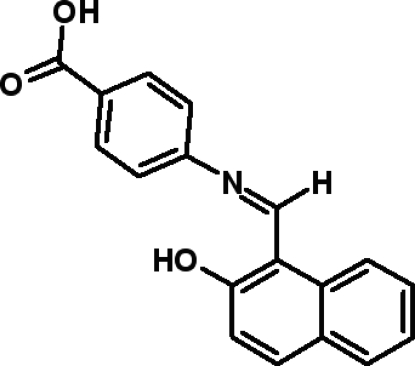


## Experimental

### 

#### Crystal data

C_18_H_13_NO_3_
*M* *_r_* = 291.29
Monoclinic, 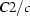

*a* = 14.7490 (12) Å
*b* = 4.9850 (4) Å
*c* = 36.750 (3) Å
β = 91.305 (1)°
*V* = 2701.3 (4) Å^3^
*Z* = 8
Mo *K*α radiation
μ = 0.10 mm^−1^
*T* = 150 (2) K
0.31 × 0.19 × 0.09 mm

#### Data collection

Bruker APEXII CCD diffractometer
Absorption correction: multi-scan (*SADABS*; Bruker, 2005 ▶) *T*_min_ = 0.970, *T*_max_ = 0.991
13445 measured reflections
3520 independent reflections
2761 reflections with *I* > 2σ(*I*)
*R*_int_ = 0.031

#### Refinement

*R*[*F* ^2^ > 2σ(*F* ^2^)] = 0.050
*wR*(*F* ^2^) = 0.145
*S* = 1.07
3520 reflections
210 parameters
H-atom parameters constrained
Δρ_max_ = 0.59 e Å^−3^
Δρ_min_ = −0.33 e Å^−3^

### 

Data collection: *APEX2* (Bruker, 2005 ▶); cell refinement: *APEX2*; data reduction: *SAINT* (Bruker, 2005 ▶); program(s) used to solve structure: *SIR97* (Altomare *et al.*, 1999 ▶); program(s) used to refine structure: *SHELXL97* (Sheldrick, 2008 ▶); molecular graphics: *ORTEP-3 for Windows* (Farrugia, 1997 ▶); software used to prepare material for publication: *WinGX* (Farrugia, 1999 ▶).

## Supplementary Material

Crystal structure: contains datablocks global, I. DOI: 10.1107/S1600536808006107/hb2705sup1.cif

Structure factors: contains datablocks I. DOI: 10.1107/S1600536808006107/hb2705Isup2.hkl

Additional supplementary materials:  crystallographic information; 3D view; checkCIF report

## Figures and Tables

**Table 1:** Hydrogen-bond geometry (Å, °)

*D*—H⋯*A*	*D*—H	H⋯*A*	*D*⋯*A*	*D*—H⋯*A*
O1—H*O*1⋯N1	0.82	1.82	2.5572 (16)	148
O2—H*O*2⋯O3^i^	0.82	1.81	2.6281 (14)	171
C14—H14⋯O2^ii^	0.93	2.56	3.3611 (17)	145
C16—H16⋯O1^iii^	0.93	2.49	3.1542 (19)	128

Symmetry codes: (i) 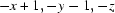; (ii) 
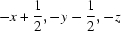
; (iii) 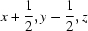.

**Fig. 1.**
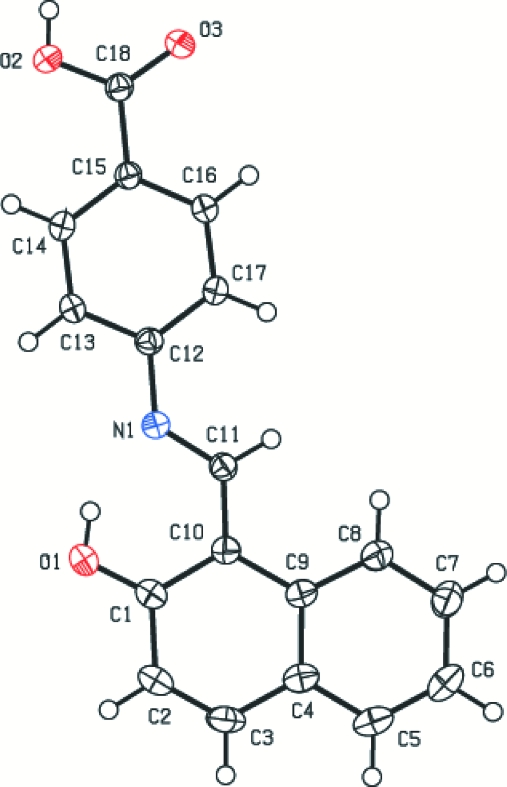
The molecular structure of (I), with displacement ellipsoids for the non-H atoms are drawn at the 50% probability level.

**Fig. 2.**
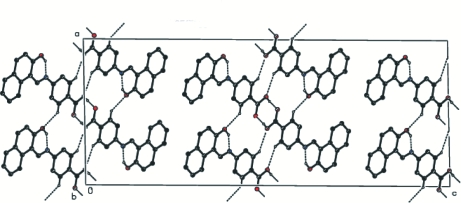
The packing for (I) showing hydrogen bonds as dashed lines.

## supplementary crystallographic information

### Comment

2-Hydroxy Schiff base ligands and their complexes, derived from the reaction of salicylaldehyde and 2-hydroxy-1-naphthaldehyde with amines are of interest due to the existence of (O—H ···N and N—H ···O) type hydrogen bonds and tautomerism between the enol-imine and keto-enamine forms. Tautomerism in 2-hydroxy Schiff bases both in solution and in the solid state was investigated using different spectroscopic techniques (Asiri & Badahdah, 2007).

In the title compound, (I), the molecule is almost planar (Fig. 1). The maximum deviation of the non-H atoms from their mean plane is 0.087 (1) Å for O3. The dihedral angle between the naphthalene ring and the benzene ring is 4.04 (6)°.

The molecular conformation is stabilized by an intramolecular O—H···N hydrogen bond (Table 1). Then, classical inversion dimers are formed by head-to-head O—H···O linkages of the carboxylic acid groups. Finally, C—H ···O interactions link the dimers into sheets (Fig. 2).

### Experimental

A solution of 4-aminobenzoic acid (5.0 g, 36.5 mmol) in hot ethanol was mixed with an ethanolic solution of 2-hydroxynaphthaldehyde (7.23 g, 36.5 mmol) and the resulting mixture was refluxed for 3 h. The mxiture was cooled to recover the crude product. Orange laths of (I) were recrystalized from ethanol. IR ν (cm^-1^); 1683.3 (C?O), 1588.1 (C?N), 1427.1 (C?C), 1301.4 (C—O) and 1152 (C—N). [*M*.p.: > 573 K, yield: 54.7%].

### Refinement

The H atoms were positioned geometrically (C—H = 0.93 Å, O—H = 0.82 Å) and refined as riding with with *U*_iso_(H) = 1.2*U*_eq_(C) or 1.5*U*_eq_(O).

### Crystal data

**Table:**

C_18_H_13_NO_3_	*F*_000_ = 1216
*M**_r_* = 291.29	*D*_x_ = 1.433 Mg m^−^^3^
Monoclinic, *C*2/*c*	Mo *K*α radiation λ = 0.71069 Å
Hall symbol: -C 2yc	Cell parameters from 3973 reflections
*a* = 14.7490 (12) Å	θ = 2.2–28.8º
*b* = 4.9850 (4) Å	µ = 0.10 mm^−^^1^
*c* = 36.750 (3) Å	*T* = 150 (2) K
β = 91.305 (1)º	Lath, orange
*V* = 2701.3 (4) Å^3^	0.31 × 0.19 × 0.09 mm
*Z* = 8	

### Data collection

**Table:**

Bruker APEXII CCD diffractometer	3520 independent reflections
Radiation source: sealed tube	2761 reflections with *I* > 2σ(*I*)
Monochromator: graphite	*R*_int_ = 0.031
*T* = 150(2) K	θ_max_ = 28.9º
φ and ω scans	θ_min_ = 2.2º
Absorption correction: multi-scan(SADABS; Bruker, 2005)	*h* = −20→19
*T*_min_ = 0.970, *T*_max_ = 0.991	*k* = −6→6
13445 measured reflections	*l* = −49→49

### Refinement

**Table:**

Refinement on *F*^2^	Secondary atom site location: difference Fourier map
Least-squares matrix: full	Hydrogen site location: inferred from neighbouring sites
*R*[*F*^2^ > 2σ(*F*^2^)] = 0.050	H-atom parameters constrained
*wR*(*F*^2^) = 0.145	*w* = 1/[σ^2^(*F*_o_^2^) + (0.0792*P*)^2^ + 1.0642*P*] where *P* = (*F*_o_^2^ + 2*F*_c_^2^)/3
*S* = 1.07	(Δ/σ)_max_ < 0.001
3520 reflections	Δρ_max_ = 0.59 e Å^−^^3^
210 parameters	Δρ_min_ = −0.33 e Å^−^^3^
Primary atom site location: structure-invariant direct methods	Extinction correction: none

### Special details

**Table:**

Geometry. Bond distances, angles *etc*. have been calculated using the rounded fractional coordinates. All su's are estimated from the variances of the (full) variance-covariance matrix. The cell e.s.d.'s are taken into account in the estimation of distances, angles and torsion angles
Refinement. Refinement on *F*^2^ for ALL reflections except those flagged by the user for potential systematic errors. Weighted *R*-factors *wR* and all goodnesses of fit *S* are based on *F*^2^, conventional *R*-factors *R* are based on *F*, with *F* set to zero for negative *F*^2^. The observed criterion of *F*^2^ > σ(*F*^2^) is used only for calculating -*R*-factor-obs *etc*. and is not relevant to the choice of reflections for refinement. *R*-factors based on *F*^2^ are statistically about twice as large as those based on *F*, and *R*-factors based on ALL data will be even larger.

### Fractional atomic coordinates and isotropic or equivalent isotropic displacement parameters (Å^2^)

**Table:**

	*x*	*y*	*z*	*U*_iso_*/*U*_eq_	
O1	0.11588 (7)	0.8430 (3)	0.11623 (4)	0.0398 (4)	
O2	0.39188 (7)	−0.3435 (2)	0.00066 (3)	0.0295 (3)	
O3	0.52323 (6)	−0.2551 (2)	0.03002 (3)	0.0277 (3)	
N1	0.26607 (8)	0.6069 (2)	0.10400 (3)	0.0232 (3)	
C1	0.15748 (10)	0.9891 (3)	0.13926 (4)	0.0293 (4)	
C2	0.10809 (11)	1.1871 (4)	0.15946 (5)	0.0387 (5)	
C3	0.14890 (11)	1.3471 (3)	0.18415 (5)	0.0368 (5)	
C4	0.24458 (10)	1.3336 (3)	0.19194 (4)	0.0273 (4)	
C5	0.28528 (12)	1.5100 (3)	0.21737 (4)	0.0333 (4)	
C6	0.37653 (13)	1.5035 (3)	0.22449 (4)	0.0367 (5)	
C7	0.43000 (12)	1.3207 (3)	0.20591 (5)	0.0382 (5)	
C8	0.39165 (11)	1.1460 (3)	0.18077 (4)	0.0328 (4)	
C9	0.29754 (10)	1.1446 (3)	0.17322 (4)	0.0235 (4)	
C10	0.25387 (9)	0.9658 (3)	0.14694 (4)	0.0227 (3)	
C11	0.30277 (9)	0.7702 (3)	0.12869 (4)	0.0222 (3)	
C12	0.31172 (9)	0.4082 (3)	0.08444 (4)	0.0216 (3)	
C13	0.26106 (9)	0.2595 (3)	0.05899 (4)	0.0237 (4)	
C14	0.30229 (9)	0.0589 (3)	0.03908 (4)	0.0236 (4)	
C15	0.39461 (9)	0.0041 (3)	0.04452 (4)	0.0212 (3)	
C16	0.44479 (9)	0.1537 (3)	0.07000 (4)	0.0237 (4)	
C17	0.40437 (9)	0.3544 (3)	0.08974 (4)	0.0237 (4)	
C18	0.44065 (9)	−0.2106 (3)	0.02406 (4)	0.0224 (3)	
HO1	0.15250	0.74990	0.10550	0.0600*	
H2	0.04600	1.20430	0.15520	0.053 (6)*	
HO2	0.42310	−0.45920	−0.00880	0.0440*	
H3	0.11410	1.47080	0.19660	0.046 (5)*	
H5	0.24950	1.63300	0.22950	0.041 (5)*	
H6	0.40270	1.61950	0.24150	0.045 (5)*	
H7	0.49230	1.31630	0.21050	0.058 (6)*	
H8	0.42870	1.02670	0.16860	0.037 (5)*	
H11	0.36440	0.75340	0.13410	0.0270*	
H13	0.19960	0.29500	0.05540	0.0280*	
H14	0.26850	−0.03930	0.02210	0.031 (4)*	
H16	0.50620	0.11760	0.07370	0.022 (4)*	
H17	0.43850	0.45380	0.10650	0.031 (4)*	

### Atomic displacement parameters (Å^2^)

**Table:**

	*U*^11^	*U*^22^	*U*^33^	*U*^12^	*U*^13^	*U*^23^
O1	0.0220 (5)	0.0457 (7)	0.0513 (7)	0.0052 (5)	−0.0055 (5)	−0.0177 (6)
O2	0.0250 (5)	0.0288 (5)	0.0348 (6)	−0.0002 (4)	0.0003 (4)	−0.0125 (4)
O3	0.0212 (5)	0.0280 (5)	0.0340 (5)	0.0019 (4)	0.0017 (4)	−0.0052 (4)
N1	0.0220 (5)	0.0239 (6)	0.0238 (6)	0.0009 (4)	0.0003 (4)	−0.0034 (4)
C1	0.0236 (7)	0.0307 (7)	0.0336 (8)	0.0035 (6)	0.0012 (6)	−0.0026 (6)
C2	0.0244 (7)	0.0432 (9)	0.0485 (10)	0.0085 (7)	0.0031 (7)	−0.0090 (8)
C3	0.0340 (8)	0.0371 (8)	0.0396 (9)	0.0111 (7)	0.0078 (7)	−0.0078 (7)
C4	0.0359 (8)	0.0237 (7)	0.0224 (7)	0.0030 (6)	0.0047 (5)	0.0017 (5)
C5	0.0487 (9)	0.0257 (7)	0.0257 (7)	0.0055 (7)	0.0049 (6)	−0.0032 (6)
C6	0.0533 (10)	0.0281 (7)	0.0285 (8)	−0.0041 (7)	−0.0049 (7)	−0.0053 (6)
C7	0.0379 (9)	0.0361 (8)	0.0402 (9)	−0.0015 (7)	−0.0091 (7)	−0.0079 (7)
C8	0.0319 (8)	0.0295 (7)	0.0368 (8)	0.0039 (6)	−0.0038 (6)	−0.0084 (6)
C9	0.0287 (7)	0.0204 (6)	0.0213 (6)	0.0014 (5)	0.0020 (5)	0.0011 (5)
C10	0.0233 (6)	0.0226 (6)	0.0222 (6)	0.0009 (5)	0.0012 (5)	0.0000 (5)
C11	0.0209 (6)	0.0230 (6)	0.0228 (6)	0.0005 (5)	0.0000 (5)	0.0001 (5)
C12	0.0219 (6)	0.0215 (6)	0.0214 (6)	0.0000 (5)	0.0016 (5)	−0.0002 (5)
C13	0.0182 (6)	0.0264 (7)	0.0264 (7)	0.0009 (5)	−0.0023 (5)	−0.0018 (5)
C14	0.0223 (6)	0.0246 (6)	0.0237 (7)	−0.0021 (5)	−0.0022 (5)	−0.0031 (5)
C15	0.0206 (6)	0.0210 (6)	0.0221 (6)	−0.0017 (5)	0.0018 (5)	−0.0010 (5)
C16	0.0188 (6)	0.0261 (7)	0.0263 (7)	−0.0006 (5)	−0.0001 (5)	−0.0033 (5)
C17	0.0210 (6)	0.0258 (7)	0.0243 (6)	−0.0020 (5)	−0.0016 (5)	−0.0048 (5)
C18	0.0223 (6)	0.0214 (6)	0.0235 (6)	−0.0025 (5)	0.0027 (5)	−0.0013 (5)

### Geometric parameters (Å, °)

**Table:**

O1—C1	1.265 (2)	C12—C17	1.4018 (19)
O2—C18	1.2914 (18)	C12—C13	1.397 (2)
O3—C18	1.2524 (16)	C13—C14	1.388 (2)
O1—HO1	0.8200	C14—C15	1.3987 (19)
O2—HO2	0.8200	C15—C16	1.396 (2)
N1—C12	1.4047 (18)	C15—C18	1.482 (2)
N1—C11	1.3254 (18)	C16—C17	1.379 (2)
C1—C2	1.442 (2)	C2—H2	0.9300
C1—C10	1.448 (2)	C3—H3	0.9300
C2—C3	1.341 (3)	C5—H5	0.9300
C3—C4	1.435 (2)	C6—H6	0.9300
C4—C9	1.413 (2)	C7—H7	0.9300
C4—C5	1.408 (2)	C8—H8	0.9300
C5—C6	1.366 (3)	C11—H11	0.9300
C6—C7	1.394 (2)	C13—H13	0.9300
C7—C8	1.381 (2)	C14—H14	0.9300
C8—C9	1.409 (2)	C16—H16	0.9300
C9—C10	1.454 (2)	C17—H17	0.9300
C10—C11	1.394 (2)		
			
C1—O1—HO1	109.00	C14—C15—C18	121.63 (13)
C18—O2—HO2	109.00	C15—C16—C17	120.76 (13)
C11—N1—C12	126.19 (12)	C12—C17—C16	119.83 (13)
O1—C1—C2	119.70 (14)	O3—C18—C15	119.63 (13)
C2—C1—C10	117.37 (13)	O2—C18—O3	123.39 (13)
O1—C1—C10	122.92 (14)	O2—C18—C15	116.99 (12)
C1—C2—C3	122.09 (15)	C1—C2—H2	119.00
C2—C3—C4	122.11 (15)	C3—C2—H2	119.00
C3—C4—C9	119.09 (14)	C2—C3—H3	119.00
C3—C4—C5	120.38 (14)	C4—C3—H3	119.00
C5—C4—C9	120.52 (14)	C4—C5—H5	119.00
C4—C5—C6	121.06 (14)	C6—C5—H5	119.00
C5—C6—C7	119.19 (15)	C5—C6—H6	120.00
C6—C7—C8	120.81 (16)	C7—C6—H6	120.00
C7—C8—C9	121.42 (14)	C6—C7—H7	120.00
C4—C9—C10	119.40 (13)	C8—C7—H7	120.00
C4—C9—C8	116.99 (13)	C7—C8—H8	119.00
C8—C9—C10	123.59 (13)	C9—C8—H8	119.00
C1—C10—C11	118.61 (13)	N1—C11—H11	118.00
C1—C10—C9	119.90 (13)	C10—C11—H11	118.00
C9—C10—C11	121.48 (12)	C12—C13—H13	120.00
N1—C11—C10	123.46 (12)	C14—C13—H13	120.00
N1—C12—C17	122.74 (13)	C13—C14—H14	120.00
C13—C12—C17	119.77 (13)	C15—C14—H14	120.00
N1—C12—C13	117.49 (12)	C15—C16—H16	120.00
C12—C13—C14	120.09 (12)	C17—C16—H16	120.00
C13—C14—C15	120.15 (13)	C12—C17—H17	120.00
C14—C15—C16	119.41 (13)	C16—C17—H17	120.00
C16—C15—C18	118.96 (12)		
			
C12—N1—C11—C10	179.29 (14)	C7—C8—C9—C10	179.74 (15)
C11—N1—C12—C13	−178.82 (14)	C4—C9—C10—C1	2.2 (2)
C11—N1—C12—C17	1.6 (2)	C4—C9—C10—C11	−177.85 (14)
O1—C1—C2—C3	−179.95 (18)	C8—C9—C10—C1	−176.23 (14)
C10—C1—C2—C3	0.8 (3)	C8—C9—C10—C11	3.8 (2)
O1—C1—C10—C9	178.67 (15)	C1—C10—C11—N1	1.4 (2)
O1—C1—C10—C11	−1.3 (2)	C9—C10—C11—N1	−178.55 (14)
C2—C1—C10—C9	−2.1 (2)	N1—C12—C13—C14	−179.43 (13)
C2—C1—C10—C11	177.93 (15)	C17—C12—C13—C14	0.1 (2)
C1—C2—C3—C4	0.5 (3)	N1—C12—C17—C16	179.04 (13)
C2—C3—C4—C5	178.31 (16)	C13—C12—C17—C16	−0.5 (2)
C2—C3—C4—C9	−0.5 (2)	C12—C13—C14—C15	0.3 (2)
C3—C4—C5—C6	−178.57 (15)	C13—C14—C15—C16	−0.3 (2)
C9—C4—C5—C6	0.2 (2)	C13—C14—C15—C18	179.30 (14)
C3—C4—C9—C8	177.61 (14)	C14—C15—C16—C17	−0.1 (2)
C3—C4—C9—C10	−0.9 (2)	C18—C15—C16—C17	−179.67 (14)
C5—C4—C9—C8	−1.1 (2)	C14—C15—C18—O2	0.7 (2)
C5—C4—C9—C10	−179.65 (14)	C14—C15—C18—O3	−179.25 (14)
C4—C5—C6—C7	0.7 (2)	C16—C15—C18—O2	−179.75 (13)
C5—C6—C7—C8	−0.5 (2)	C16—C15—C18—O3	0.3 (2)
C6—C7—C8—C9	−0.5 (2)	C15—C16—C17—C12	0.5 (2)
C7—C8—C9—C4	1.3 (2)		

### Hydrogen-bond geometry (Å, °)

**Table:**

*D*—H···*A*	*D*—H	H···*A*	*D*···*A*	*D*—H···*A*
O1—HO1···N1	0.82	1.82	2.5572 (16)	148
O2—HO2···O3^i^	0.82	1.81	2.6281 (14)	171
C14—H14···O2^ii^	0.93	2.56	3.3611 (17)	145
C16—H16···O1^iii^	0.93	2.49	3.1542 (19)	128

Symmetry codes: (i) −*x*+1, −*y*−1, −*z*; (ii) −*x*+1/2, −*y*−1/2, −*z*; (iii) *x*+1/2, *y*−1/2, *z*.
